# Knowledge, Awareness, and Practices Toward Irritable Bowel Syndrome (IBS) Among the General Population in the Al-Baha Region, Saudi Arabia

**DOI:** 10.7759/cureus.108208

**Published:** 2026-05-03

**Authors:** Faisal Alghamdi, Ali M Alqarni, Mohammed Ali Alzahrani, Omran M Alzahrani, Rashed Alghamdi, Anas Alalyani, Saeed Saleh Aziz Alghamdi, Saad Alghamdi, Ahmad Alghamdi, Mohammed Alghamdi, Abdulrahman A Alzahrani

**Affiliations:** 1 Department of Internal Medicine, King Fahad Hospital, Al-Baha, SAU

**Keywords:** al-baha, awareness, irritable bowel syndrome (ibs), knowledge, ksa, practice

## Abstract

Introduction

Irritable bowel syndrome (IBS) is a chronic medical condition that presents with abdominal pain, abdominal bloating, diarrhea, among others. Understanding the knowledge, awareness, and practice of IBS is crucial for the early recognition of symptoms, treatment, and management of the condition.

Methods

This was a cross-sectional, questionnaire-based study that utilized data from a sample of 421 participants drawn from the general adult population in the Al-Baha region of Saudi Arabia. Data were cleaned, coded, and then analyzed using IBM SPSS Statistics for Windows, Version 26 (Released 2018; IBM Corp., Armonk, NY, USA), to derive key insights.

Results

The study found that 85 (20.2%) had high knowledge, the majority 299 (71.0%) had medium knowledge, while only 37 participants (8.8%) had low knowledge about IBS. Participants aged 20-39 years (p < 0.001), those with postgraduate studies (p = 0.039), and those employed (p = 0.002) demonstrated considerably good knowledge about the condition compared to other groups. The most frequently reported symptom of IBS was abdominal bloating (368, 87.4%); psychological stress (388, 92.2%) was the most commonly reported factor that worsens IBS. The most common source of information about the condition was social media (214, 50.8%). Being employed was positively associated with high knowledge about IBS (AOR = 1.96; 95% CI = 1.048-3.665; p = 0.035).

Conclusion

The study found the level of knowledge about IBS to be inadequate among the population, with significant gaps in understanding the potential health risks posed by the condition. The most common source of information about IBS was social media. Being employed was significantly associated with high knowledge of the condition. The study highlights the need for targeted health education and awareness campaigns to increase awareness of the condition, ultimately improving treatment outcomes.

## Introduction

Irritable bowel syndrome (IBS) is a non-organic disorder of gut-brain interaction (DGBI) characterized by abdominal pain and altered bowel habits [1]. The prevalence of IBS varies across regions, with studies showing that it ranges from 10% to 25% in the United States, up to 11.5% in Europe, and up to 20.87% in the Middle East [2-4]. In Saudi Arabia, reported rates range from 16.3% to over 30% [5], highlighting the burden of the disorder in the country. Although the condition affects people of all ages, it is more prevalent among women and individuals aged below 35, with prevalence declining significantly after the age of 50 [2]. Apart from young age and female gender, other risk factors include stress, unhealthy lifestyle choices, smoking, and family history [6]. Currently, there are limited effective treatment options for IBS, which makes it a major healthcare issue that significantly affects patients’ quality of life.

IBS significantly increases healthcare costs, especially given that between 20% and 50% of patients attend gastrointestinal clinics [7]. Additionally, more than 90% of patients experience at least one comorbidity, with common conditions including anxiety, depression, fibromyalgia, chronic fatigue syndrome, sleep disturbances, migraine, and chronic pelvic pain [8]. Despite the health challenges associated with IBS, most individuals either do not seek medical care or are unaware of appropriate self-management strategies [9]. Furthermore, diagnosis of IBS can be challenging because it lacks specific biochemical or biological markers, and its symptoms vary widely among patients [1]. These factors make IBS a challenging condition for both patients and healthcare providers, highlighting the need for adequate knowledge of the condition in both groups.

It is important for the general population to have adequate knowledge of IBS because such knowledge facilitates effective self-management and preventive lifestyle practices. With sufficient awareness, early diagnosis and proper care can be achieved, leading to better patient outcomes. Therefore, it is also important to assess the level of knowledge in the population in order to guide targeted interventions. While similar studies, as well as others on the prevalence of IBS, have been conducted previously, there has been limited research focusing on the Al-Baha region of Saudi Arabia. Thus, this study seeks to investigate knowledge, awareness, and practices regarding IBS among the general population in the Al-Baha region.

## Materials and methods

Study design and setting

A descriptive, cross-sectional study was conducted to assess knowledge, awareness, and practices toward IBS among the general adult population in the Al-Baha region of Saudi Arabia. The study was carried out across the Al-Baha region using an electronic questionnaire distributed to community residents. Data collection took place over a six-month period, from July 1, 2025 to January 1, 2026.

Study population and sampling

The target population for this study consisted of adult residents (≥18 years) of the Al-Baha region, Saudi Arabia. Participants were recruited using a non-probability convenience sampling technique through online distribution of the questionnaire via social media platforms. The survey specifically targeted members of the general public residing in the region, rather than hospital-based participants. Healthcare professionals were excluded to minimize potential knowledge bias. Individuals with cognitive impairments that could affect their ability to understand or complete the questionnaire were also excluded.

Sample size calculation

The sample size was calculated using the Raosoft® Sample Size Calculator (Raosoft, Inc., Seattle, WA, USA). The calculation was based on a 95% confidence level, a margin of error of 5%, and an assumed population proportion of 50% to obtain the maximum required sample size. Based on these parameters, the minimum required sample size was estimated to be 385 participants. However, to improve reliability, the sample size was increased to 421 respondents.

Data collection tool and technique

We collected data using a structured electronic questionnaire distributed through Google Forms (Google, Inc., Mountain View, CA, USA), which was adapted from a previous cross-sectional study conducted in Aseer, Saudi Arabia, that assessed public awareness of IBS [10]. The survey was administered in Arabic. Experts in public health and gastroenterology reviewed the questionnaire to ensure clarity and adequate coverage of all relevant topics. Prior to launching the main survey, we conducted a pilot test with approximately 5%-10% of the target sample to assess reliability and ease of understanding. Responses from the pilot were not included in the final analysis.

The questionnaire included questions on sociodemographic characteristics, participants’ awareness and beliefs about IBS, knowledge (addressing the definition, symptoms, risk factors, diagnosis, and common misconceptions about IBS), and practices and health-seeking behaviors (see Appendix 1). In the knowledge section, participants received 1 point for each correct answer and 0 points for incorrect or “I don’t know” responses. These were then summed to create an overall knowledge score, which was categorized as low, medium, or high. Each correct response in the knowledge section was assigned 1 point, while incorrect and “I don’t know” responses were assigned 0. The total knowledge score was calculated by summing all responses, with higher scores indicating better knowledge. Scores were then converted into percentages and categorized as low (<50%), medium (50%-75%), and high (>75%).

The survey was distributed online, primarily through social media platforms, targeting residents of the Al-Baha region. Participation was voluntary, and all participants provided electronic informed consent before beginning the survey.

Statistical analysis

We exported the data and entered it into Microsoft Excel (Microsoft® Corp., Redmond, WA, USA) for coding and cleaning, which involved checking for completeness, consistency, and missing values. Once the data were cleaned, we transferred the dataset to IBM SPSS Statistics for Windows, Version 26 (Released 2018; IBM Corp., Armonk, NY, USA), for analysis. Descriptive statistics were used to summarize the data, with categorical variables reported as frequencies and percentages. A composite knowledge score was generated and categorized into different levels.

To assess the association between knowledge level and sociodemographic factors, chi-square tests were performed. Variables with a p-value < 0.05 in the bivariate analysis were included in a multivariate logistic regression model to identify independent predictors of high knowledge. Results were presented as adjusted odds ratios (AORs) with 95% confidence intervals (CIs). A p-value < 0.05 was considered statistically significant.

Ethical considerations

Ethical approval for the study was obtained from the Scientific Research Committee, Al-Baha Health Cluster (Approval No. BHC-28012026-4), prior to data collection. The study followed socially acceptable research practices and ensured the confidentiality of all collected data. Participation was voluntary, and written informed consent was obtained from all participants. Clear communication was maintained between the researchers and participants throughout the study.

## Results

Table 1 shows that a total of 421 participants were included in the study. Nearly half of the participants, 180 (42.8%), were aged 20-39 years; the majority, 270 (64.1%), were males; and the vast majority were married (303, 72.0%). Additionally, the vast majority, 295 (70.1%), had postgraduate education, and more than half, 236 (56.1%), were employed.

**Table 1:** Demographic characteristics of participants (N = 421) Demographic data presented in frequencies (n) and proportions (%).

Demographics	Category	N (%)
Age (years)	18-20 years	25 (5.9%)
	20-39	180 (42.8%)
	40-59	171 (40.6%)
	Greater than 60	45 (10.7%)
Gender	Female	151 (35.9%)
	Male	270 (64.1%)
Marital status	Married	303 (72.0%)
	Single	118 (28.0%)
Education level	Middle (intermediate) education	19 (4.5%)
	Post-graduate studies	295 (70.1%)
	Primary	8 (1.9%)
	Secondary	99 (23.5%)
Employment status	Employee	236 (56.1%)
	Unemployed	185 (43.9%)

The disease awareness of IBS showed that an overwhelming majority, 408 (96.9%), of the participants had heard about IBS, with more than half, 228 (54.2%), perceiving their level of awareness about the condition to be good (Table 2). The common sources of information about the condition were social media (214, 50.8%) and friends (197, 46.8%). The majority of the participants correctly reported that IBS is a common disease (350, 83.1%) and that the disease can significantly affect a person’s life (368, 87.4%). Interestingly, only 55 participants (13.1%) were aware that IBS cannot cause permanent damage to the intestines. Nearly one-third of the participants, 142 (33.7%), had received education or awareness about IBS.

**Table 2:** Disease awareness Disease awareness presented in frequencies (n) and proportions (%).

Questions	Category	N (%)
Have you ever heard of irritable bowel syndrome (IBS)?	No	13 (3.1%)
	Yes	408 (96.9%)
How do you assess your level of awareness of irritable bowel syndrome?	Excellent	123 (29.2%)
	Good	228 (54.2%)
	Weak	70 (16.6%)
What is the source of your information about this disease?	Social media	214 (50.8%)
	Doctors	186 (44.2%)
	Medical websites	150 (35.6%)
	Television	53 (12.6%)
	Friends	197 (46.8%)
Do you think irritable bowel syndrome (IBS) is a common disease?	I don’t know	43 (10.2%)
	No	28 (6.7%)
	Yes	350 (83.1%)
Do you think irritable bowel syndrome (IBS) can significantly affect a person’s life?	I don’t know	29 (6.9%)
	No	24 (5.7%)
	Yes	368 (87.4%)
Do you think irritable bowel syndrome (IBS) is underdiagnosed?	I don’t know	88 (20.9%)
	No	37 (8.8%)
	Yes	296 (70.3%)
Is irritable bowel syndrome (IBS) a chronic disease?	I don’t know	95 (22.6%)
	No	70 (16.6%)
	Yes	256 (60.8%)
Do you think society is very aware of irritable bowel syndrome?	I don’t know	68 (16.2%)
	No	305 (72.4%)
	Yes	48 (11.4%)
Does irritable bowel syndrome cause permanent damage to the intestines?	I don’t know	172 (40.9%)
	No	55 (13.1%)
	Yes	194 (46.1%)
Have you ever received education or awareness about IBS?	No	279 (66.3%)
	Yes	142 (33.7%)

Knowledge about IBS revealed that the overwhelming majority, 385 (91.4%), knew the correct definition of IBS (Table 3). The frequently reported symptoms of IBS were abdominal bloating (368, 87.4%) and recurrent abdominal pain (290, 68.9%); psychological stress (388, 92.2%) was the most commonly reported factor that worsens IBS. More than half of the participants were aware that the condition is diagnosed based on clinical symptoms (245, 58.2%), while about half, 210 (49.9%), were aware that unintentional weight loss is not consistent with IBS symptoms.

**Table 3:** Knowledge Knowledge presented in frequencies (n) and proportions (%).

Questions	Category	N (%)
Irritable bowel syndrome (IBS) is:	Functional disorder of the digestive system	385 (91.4%)
	Infectious disease	2 (0.5%)
	Intestinal inflammation	34 (8.1%)
Which of these symptoms usually appear with irritable bowel syndrome?	Recurrent abdominal pain	290 (68.9%)
	Abdominal bloating	368 (87.4%)
	Diarrhea or constipation	209 (49.6%)
	Rectal bleeding	36 (8.6%)
Which of these factors worsens the symptoms of irritable bowel syndrome?	Psychological stress	388 (92.2%)
	Nutritional factors	300 (71.3%)
	Intestinal inflammation	106 (25.2%)
	Medications	105 (24.9%)
	Smoking	147 (34.9%)
Irritable bowel syndrome (IBS) is diagnosed by:	Clinical symptoms	245 (58.2%)
	Imaging X-rays	62 (14.7%)
	Laboratory tests	110 (26.1%)
	Surgical operations	4 (1.0%)
Which of these symptoms is not consistent with irritable bowel syndrome (IBS)	Abdominal pain subsides after defecation	171 (40.6%)
	Unintentional weight loss	210 (49.9%)
	Abdominal bloating	81 (19.2%)
	Diarrhea and constipation	52 (12.4%)
Irritable bowel syndrome (IBS) is considered:	A chronic medical condition	331 (78.6%)
	An acute inflammatory condition	162 (38.5%)
	A surgery emergency	7 (1.7%)
	An infectious disease	8 (1.9%)
Adhering to a diet improves symptoms of irritable bowel syndrome	Correct	353 (83.8%)
	I don’t know	55 (13.1%)
	Incorrect	13 (3.1%)
Psychological stress increases the symptoms of irritable bowel syndrome	Correct	408 (96.9%)
	I don’t know	11 (2.6%)
	Incorrect	2 (0.5%)
Depression is linked to irritable bowel syndrome	Correct	297 (70.5%)
	I don’t know	95 (22.6%)
	Incorrect	29 (6.9%)
Irritable bowel syndrome increases the likelihood of developing bowel cancer	Correct	127 (30.2%)
	I don’t know	245 (58.2%)
	Incorrect	49 (11.6%)
Which of the following symptoms is considered a warning sign that requires visiting a doctor?	Unintentional weight loss	232 (55.1%)
	Rectal bleeding	314 (74.6%)
	Persistent abdominal bloating	161 (38.2%)
	Persistent diarrhea	192 (45.6%)
	Persistent constipation	175 (41.6%)

The majority of participants were aware that adhering to a diet improves symptoms (353, 83.8%), that psychological stress increases symptoms (408, 96.9%), and that depression is linked to IBS (297, 70.5%). However, the study found significant knowledge gaps, with only 49 participants (11.6%) knowing that IBS does not increase the likelihood of developing bowel cancer.

Approximately three-quarters of participants, 331 (78.6%), were aware that IBS is a chronic medical condition and that rectal bleeding (314, 74.6%) is a warning sign requiring medical consultation.

The results in Table 4 show that the common IBS treatments were stress management (356, 84.6%) and dietary modification (280, 66.5%). About one-third of the participants, 168 (39.9%), expressed their likelihood of seeking medical advice in case of persistent abdominal discomfort, while less than half, 180 (42.8%), reported having consulted a doctor due to recurring bowel-related symptoms. A notable proportion, 182 (43.2%), believed that traditional herbal medicine is more effective than conventional medication in treating IBS symptoms. Nearly half, 207 (49.2%), of the participants reported practicing stress management strategies to alleviate bowel symptoms.

**Table 4:** Practice Practice presented in frequencies (n) and proportions (%).

Questions	Category	N (%)
How is irritable bowel syndrome (IBS) treated?	Stress management	356 (84.6%)
	Dietary modifications	280 (66.5%)
	Medications	228 (54.2%)
Can irritable bowel syndrome be diagnosed via lower endoscopy	Correct	165 (39.2%)
	I don’t know	196 (46.6%)
	Incorrect	60 (14.3%)
If you are experiencing persistent abdominal discomfort or a change in bowel habits, how likely are you to seek medical advice?	I don’t think so	60 (14.3%)
	Maybe	193 (45.8%)
	Mostly	168 (39.9%)
Have you ever consulted a doctor about recurring bowel-related symptoms?	No	241 (57.2%)
	Yes	180 (42.8%)
Do you believe that traditional herbal medicine is more effective than medications in treating irritable bowel syndrome symptoms?	I don’t know	119 (28.3%)
	No	120 (28.5%)
	Yes	182 (43.2%)
Have you ever modified eating habits because of digestive symptoms?	No	101 (24.0%)
	Yes	320 (76.0%)
Do you regularly identify and avoid foods that cause bowel-related symptoms?	No	123 (29.2%)
	Yes	298 (70.8%)
Are you currently following any specific diet to control your bowel symptoms?	No	257 (61.0%)
	Yes	164 (39.0%)
Do you practice stress management strategies to alleviate bowel symptoms?	No	214 (50.8%)
	Yes	207 (49.2%)
Are you using over-the-counter medications to treat your bowel symptoms?	No	254 (60.3%)
	Sometimes	108 (25.7%)
	Yes	59 (14.0%)
Have bowel-related symptoms ever caused you to miss work, school, or important commitments?	No	253 (60.1%)
	Yes	168 (39.9%)
Have you ever been diagnosed with irritable bowel syndrome by a healthcare professional?	No	320 (76.0%)
	Yes	101 (24.0%)
If you are diagnosed with IBS, will you adhere to the prescribed plans and follow-up appointments?	No	28 (27.7%)
	Yes	73 (72.3%)
Irritable bowel syndrome (IBS) is a real medical condition that requires treatment	Correct	359 (85.3%)
	I don’t know	54 (12.8%)
	Incorrect	8 (1.9%)
What is the main reason that might prevent you from seeking medical advice regarding your bowel symptoms	Fear of being diagnosed with a serious disease	175 (41.6%)
	Previous unsatisfactory experiences with doctors	127 (30.2%)
	Preference for self-treatment/herbal remedies	131 (31.1%)
	Difficulty accessing a specialist	132 (31.4%)
How long would you wait before seeking medical advice regarding persistent bowel symptoms	Less than a month	201 (47.7%)
	From 1 to 3 months	124 (29.5%)
	More than 3 months	96 (22.8%)
Have gastrointestinal symptoms ever affected your daily activities or social life?	No	136 (32.3%)
	Yes	285 (67.7%)

Interestingly, only 101 participants (24.0%) had been diagnosed with IBS by a healthcare professional, while nearly three-quarters, 73 (72.3%), indicated that they would adhere to prescribed treatment plans and follow-up programs if diagnosed with IBS.

The most common reason preventing participants from seeking medical care was fear of being diagnosed with a serious disease (175, 41.6%), while the majority, 285 (67.7%), reported that gastrointestinal symptoms had affected their daily activities and social life.

**Figure 1:**
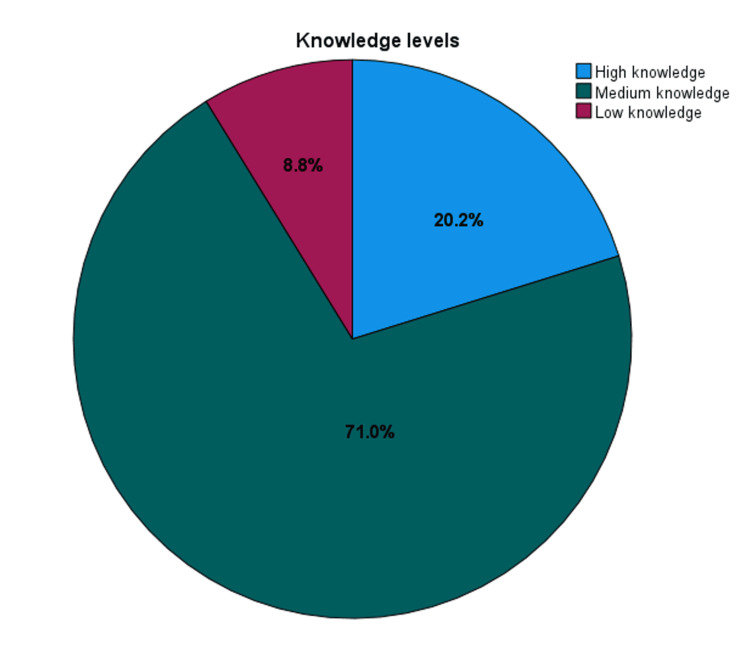
Distribution of the knowledge levels about IBS among the participants IBS: irritable bowel syndrome

Figure 1 illustrates the knowledge levels about IBS among the participants. Of the 421 participants, about 85 (20.2%) had high knowledge, the majority, 299 (71.0%), had medium knowledge, while only 37 participants (8.8%) had low knowledge.

The results revealed that participants aged 20-39 years (p < 0.001), those with postgraduate studies (p = 0.039), and those employed (p = 0.002) demonstrated considerably good knowledge about the condition compared to other groups. However, other variables were not statistically significant (p > 0.05) (Table 5).

**Table 5:** Association between participants’ attributes and knowledge about IBS * Significant at p < 0.05 level. IBS: irritable bowel syndrome

Variables	Knowledge about IBS				
	Category	High	Medium	Low	p-value
Age	Less than 20	0 (0.0%)	20 (80.0%)	5 (20.0%)	<0.001*
	20-39	56 (31.1%)	115 (63.9%)	9 (5.0%)	
	40-59	26 (15.2%)	126 (73.7%)	19 (11.1%)	
	Greater than 60	3 (6.7%)	38 (84.4%)	4 (8.9%)	
Gender	Female	23 (15.2%)	115 (76.2%)	13 (8.6%)	0.153
	Male	62 (23.0%)	184 (68.1%)	24 (8.9%)	
Marital status	Married	56 (18.5%)	218 (71.9%)	29 (9.6%)	0.294
	Single	29 (24.6%)	81 (68.6%)	8 (6.8%)	
Education level	Middle	1 (5.3%)	16 (84.2%)	2 (10.5%)	0.039*
	Post-graduate studies	72 (24.4%)	202 (68.5%)	21 (7.1%)	
	Primary	1 (12.5%)	6 (75.0%)	1 (12.5%)	
	Secondary	11 (11.1%)	75 (75.8%)	13 (13.1%)	
Employment status	Employee	62 (26.3%)	157 (66.5%)	17 (7.2%)	0.002*
	Unemployed	23 (12.4%)	142 (76.8%)	20 (10.8%)	

In the multivariate logistic regression model (Table 6), being employed was positively associated with high knowledge about IBS (AOR = 1.96; 95% CI = 1.048-3.665; p = 0.035). However, the other variables were not statistically significant.

**Table 6:** Multivariate logistic regression analysis of factors associated with high knowledge about IBS * Significant at p < 0.05 level. AOR: adjusted odds ratio; CI: confidence interval; IBS: irritable bowel syndrome

Variables	AOR	95% CI	p-value
Age (years)	1.50	0.974-2.314	0.066
Gender	1.38	0.760-2.521	0.288
Marital status	1.09	0.587-2.035	0.779
Education level	1.53	0.880-2.641	0.132
Employment status	1.96	1.048-3.665	0.035*

## Discussion

IBS is a significant health challenge leading to problems with food movement through the digestive tract. Despite growing concerns about IBS in Saudi Arabia, there is limited data on the knowledge, awareness, and practice of the condition in the country [11,12]. This study aimed to bridge this gap by assessing knowledge, awareness, and practice of IBS in the Al-Baha region of Saudi Arabia.

The study revealed significant gaps in knowledge and awareness of IBS, as demonstrated by the majority of participants who had moderate to low knowledge, with only about 85 participants (20.2%) having high knowledge. This finding is consistent with that of a Saudi study conducted by Baig et al., which reported about 19.2% high knowledge of IBS among the Saudi community [6]. These gaps were further evidenced by the findings that only 55 participants (13.1%) were aware that IBS cannot cause permanent damage to the intestines, and only 49 participants (11.6%) understood that the condition does not increase the likelihood of developing bowel cancer.

The findings highlight the need for enhanced public health interventions and targeted education programs to increase awareness of IBS in the population.

The study observed that participants who were employed demonstrated higher knowledge of the condition (p = 0.002), with employment status (AOR = 1.96; 95% CI = 1.048-3.665; p = 0.035) being a significant predictor of high knowledge, likely due to greater exposure to information about IBS in the workplace, underscoring the role of relevant professional experience in driving learning and understanding of medical conditions.

Participants aged 20-39 years demonstrated significantly higher knowledge about IBS (p < 0.001) compared with older adults, consistent with the Saudi study conducted by Bawahab et al., which found higher knowledge about IBS among individuals aged 21-30 years [10]. Similarly, the findings by von dem Knesebeck et al. also reported higher knowledge levels among young adults aged 18-40 years [13], likely due to increased literacy levels and greater exposure to digital health information among the adult population in Saudi Arabia. Additionally, significantly higher knowledge was observed among participants with postgraduate studies, aligning with the study conducted by Kareem and Sahib, who reported that participants with a Master’s degree or higher exhibited greater knowledge about IBS [14], emphasizing the significance of advanced education in enhancing knowledge and understanding of medical conditions.

The most frequently reported symptom of IBS was abdominal bloating (368, 87.4%), while psychological stress (388, 92.2%) was the most commonly reported factor that worsens IBS. These findings are in line with those of a study by Hod et al., which reported abdominal bloating as a common symptom associated with anxiety and depression in IBS patients [15]. The most common source of information about the condition was social media (214, 50.8%), highlighting the growing use of digital platforms in disseminating health information to the public.

The most common barrier preventing participants from seeking medical care was the fear of being diagnosed with a serious disease (175, 41.6%), with only 101 participants (24.0%) reporting having been diagnosed with IBS by a healthcare professional, reflecting low uptake of medical intervention in the population. However, nearly three-quarters of participants, 309 (73.4%), indicated that they would adhere to prescribed plans and follow-up programs if diagnosed with IBS, underscoring a positive attitude and willingness to comply with medical recommendations.

The limitations of the study that should be considered when interpreting the findings include the cross-sectional design, which allows identification of associations between variables but not causality. Convenience sampling may have introduced bias by overrepresenting some groups and excluding others. Furthermore, the findings cannot be generalized to other populations, as the study was conducted in a single region, the Al-Baha region. Additionally, data on participants’ nationality were not collected, which may limit the generalizability of the findings across different population groups. The study also relied on anonymous, self-administered responses, which may introduce response bias. Although anonymity, lack of incentives, and absence of time pressure may have reduced social desirability bias and the likelihood of consulting external sources, this possibility cannot be entirely excluded. These limitations should be considered when interpreting the results.

## Conclusions

The study found the level of knowledge about IBS to be inadequate among the population, with significant gaps in understanding the potential health risks posed by the condition. The most common source of information about IBS was social media. Being employed was significantly associated with high knowledge of the condition. The study highlights the need for targeted health education and awareness campaigns to increase awareness of the condition, ultimately improving treatment outcomes.

## Appendices

Appendix 1. Survey on irritable bowel syndrome (IBS)

Participant Information

Consent Form for Research Participation

Research Title: Knowledge, Awareness, and Attitudes Toward Irritable Bowel Syndrome Among the General Population in Al-Baha Region, Saudi Arabia

You are invited to participate in this study, which aims to assess community awareness and knowledge regarding Irritable Bowel Syndrome (IBS).

Your participation is voluntary. All responses will be handled with strict confidentiality and will be used only for scientific research purposes.

By agreeing to participate, you confirm the following:

- Your participation is voluntary, and you may withdraw at any time.

- All information will remain confidential and will not contain any identifying data.

* Indicates a required question.

1. I agree to participate in this study.

Select one option only.

( ) Yes

( ) No

Personal Information

2. Age

Select one option only.

( ) Less than 20 years ( ) 20-39 years

( ) 40-59 years

( ) More than 60 years

3. Gender

Select one option only.

( ) Male

( ) Female

4. Marital status

Select one option only.

( ) Single

( ) Married

5. Educational level

Select one option only.

( ) Primary education

( ) Intermediate education ( ) Secondary education

( ) Postgraduate studies

6. Employment status

Select one option only.

( ) Employed

( ) Unemployed

Disease Awareness Section

7. Have you heard of Irritable Bowel Syndrome before?

Select one option only.

( ) Yes

( ) No

8. How would you rate your level of awareness about Irritable Bowel Syndrome?

Select one option only.

( ) Excellent ( ) Good

( ) Poor

9. What is your source of information about this disease?

Select all applicable options.

( ) Social media ( ) Physicians

( ) Medical websites ( ) Television

( ) Friends

10. Do you think Irritable Bowel Syndrome is a common disease?

Select one option only.

( ) Yes

( ) No

( ) I do not know

11. Do you think Irritable Bowel Syndrome can significantly affect a person's life?

Select one option only.

( ) Yes

( ) No

( ) I do not know

12. Do you think Irritable Bowel Syndrome is underdiagnosed?

Select one option only.

( ) Yes

( ) No

( ) I do not know

13. Is Irritable Bowel Syndrome a chronic condition?

Select one option only.

( ) Yes

( ) No

( ) I do not know

14. Do you think the community is highly aware of Irritable Bowel Syndrome?

Select one option only.

( ) Yes

( ) No

( ) I do not know

15. Does Irritable Bowel Syndrome cause permanent damage to the intestines?

Select one option only.

( ) Yes

( ) No

( ) I do not know

16. Have you ever received health education about this disease?

Select one option only.

( ) Yes

( ) No

Knowledge Section

Please select the best answer.

17. Irritable Bowel Syndrome is:

Select one option only.

( ) A functional gastrointestinal disorder ( ) Inflammation of the intestines

( ) An infectious disease ( ) A cancerous condition

18. Which of the following symptoms are commonly associated with Irritable Bowel Syndrome?

Select all applicable options.

( ) Recurrent abdominal pain ( ) Abdominal bloating

( ) Diarrhea or constipation ( ) Rectal bleeding

19. Which of the following factors may worsen Irritable Bowel Syndrome symptoms?

Select all applicable options.

( ) Psychological factors ( ) Dietary factors

( ) Gastrointestinal infections ( ) Medications

( ) Smoking

20. Irritable Bowel Syndrome is diagnosed based on:

Select one option only.

( ) Clinical symptoms ( ) Laboratory tests

( ) Imaging studies

( ) Surgical procedures

21. Which of the following symptoms is not typical of Irritable Bowel Syndrome?

Select all applicable options.

( ) Abdominal pain relieved by defecation ( ) Unintentional weight loss

( ) Abdominal bloating

( ) Diarrhea and constipation

22. Irritable Bowel Syndrome is considered:

Select all applicable options.

( ) A chronic medical condition

( ) Acute intestinal inflammation ( ) A surgical emergency

( ) An infectious condition

23. Adherence to a specific diet improves Irritable Bowel Syndrome symptoms.

Select one option only.

( ) True ( ) False

( ) I do not know

24. Psychological stress increases Irritable Bowel Syndrome symptoms.

Select one option only.

( ) True ( ) False

( ) I do not know

25. Depression is associated with Irritable Bowel Syndrome.

Select one option only.

( ) True ( ) False

( ) I do not know

26. Irritable Bowel Syndrome increases the risk of intestinal cancer.

Select one option only.

( ) True ( ) False

( ) I do not know

27. Which of the following symptoms is considered an alarm symptom that requires medical evaluation?

Select all applicable options.

( ) Unintentional weight loss ( ) Rectal bleeding

( ) Persistent abdominal bloating ( ) Persistent diarrhea

( ) Persistent constipation

28. How is Irritable Bowel Syndrome treated?

Select all applicable options.

( ) Stress management ( ) Dietary modification ( ) Medications

29. Irritable Bowel Syndrome can be diagnosed using lower gastrointestinal endoscopy.

Select one option only.

( ) True ( ) False

( ) I do not know

30. If you experience persistent abdominal discomfort or a change in bowel habits, how likely are you to seek medical advice?

Select one option only.

( ) Likely ( ) Maybe

( ) Unlikely

31. Have you ever consulted a physician regarding recurrent bowel-related symptoms?

Select one option only.

( ) Yes

( ) No

32. Do you think traditional herbal medicine, such as cumin, mint, anise, and honey, is more effective than medications in treating Irritable Bowel Syndrome symptoms?

Select one option only.

( ) Yes

( ) No

( ) I do not know

Practice Section

33. Have you ever modified your dietary habits because of gastrointestinal symptoms?

Select one option only.

( ) Yes

( ) No

34. Do you regularly identify and avoid foods that trigger bowel-related symptoms?

Select one option only.

( ) Yes

( ) No

35. Are you currently following any specific diet to control bowel symptoms?

Select one option only.

( ) Yes

( ) No

36. Do you practice stress-management strategies to relieve bowel symptoms?

Select one option only.

( ) Yes

( ) No

37. Do you use over-the-counter medications to treat bowel symptoms?

Select one option only.

( ) Yes

( ) No

( ) Sometimes

38. Have bowel-related symptoms ever caused you to miss work, school, or important commitments?

Select one option only.

( ) Yes

( ) No

39. Have you ever been diagnosed with Irritable Bowel Syndrome by a healthcare professional?

Select one option only.

( ) Yes

( ) No

40. If you were diagnosed with Irritable Bowel Syndrome, would you adhere to the prescribed treatment plan and follow-up appointments?

Select one option only.

( ) Yes

( ) No

41. Irritable Bowel Syndrome is a real medical condition that requires treatment.

Select one option only.

( ) True ( ) False

( ) I do not know

42. What is the main reason that may prevent you from seeking medical advice for bowel symptoms?

Select all applicable options.

( ) Fear of being diagnosed with a serious disease

( ) Previous unsatisfactory experiences with physicians ( ) Preference for self-treatment or herbal remedies

( ) Difficulty accessing a specialist

43. How long would you wait before seeking medical advice for persistent bowel symptoms?

Select one option only.

( ) Less than one month ( ) One to three months

( ) More than three months

44. Have gastrointestinal symptoms ever affected your daily activities or social life?

Select one option only.

( ) Yes

( ) No

45. Data collector number

Select one option only.

( ) 1

( ) 2

( ) 3

( ) 4

( ) 5

( ) 6

( ) 7

( ) 8

( ) 9

( ) 10

( ) 11
